# Dual-channel deep graph convolutional neural networks

**DOI:** 10.3389/frai.2024.1290491

**Published:** 2024-04-04

**Authors:** Zhonglin Ye, Zhuoran Li, Gege Li, Haixing Zhao

**Affiliations:** ^1^College of Computer, Qinghai Normal University, Xining, Qinghai, China; ^2^The State Key Laboratory of Tibetan Intelligent Information Processing and Application, Xining, Qinghai, China; ^3^Tibetan Information Processing and Machine Translation Key Laboratory of Qinghai Province, Xining, Qinghai, China; ^4^Key Laboratory of Tibetan Information Processing, Ministry of Education, Xining, Qinghai, China

**Keywords:** DeepGCN, D2GCN, graph neural networks, graph convolutional neural networks, GNNs

## Abstract

The dual-channel graph convolutional neural networks based on hybrid features jointly model the different features of networks, so that the features can learn each other and improve the performance of various subsequent machine learning tasks. However, current dual-channel graph convolutional neural networks are limited by the number of convolution layers, which hinders the performance improvement of the models. Graph convolutional neural networks superimpose multi-layer graph convolution operations, which would occur in smoothing phenomena, resulting in performance decreasing as the increasing number of graph convolutional layers. Inspired by the success of residual connections on convolutional neural networks, this paper applies residual connections to dual-channel graph convolutional neural networks, and increases the depth of dual-channel graph convolutional neural networks. Thus, a dual-channel deep graph convolutional neural network (D2GCN) is proposed, which can effectively avoid over-smoothing and improve model performance. D2GCN is verified on CiteSeer, DBLP, and SDBLP datasets, the results show that D2GCN performs better than the comparison algorithms used in node classification tasks.

## 1 Introduction

The introduction of Convolutional Neural Networks (CNNs) has brought significant improvements to these areas, such as natural language processing, video processing, and image classification. However, traditional CNNs can only process European spatial data such as image (He et al., 2016), text (Hu et al., 2014), speech (Hinton et al., 2012), etc. A non-European spatial data, which is graph data, has attracted much attention for its ubiquity. Many applications in real life can be naturally represented by graph data, such as transportation networks, worldwide webs, and social networks, etc. How to define CNNs on graph data is receiving more and more attention. Drawing on the modeling ability of CNNs to local structures and the ubiquitous dependencies in graph data, Graph Convolutional Networks (GCNs) became one of the most active research fields. With the wide application of GCNs in data mining, such as recommendation system (Monti et al., 2017a; Ying et al., 2018) and point cloud segmentation (Li et al., 2019; Wang et al., 2019), researchers will pay more attention to the improvement of GCNs performance.

Another key factor behind the success of CNNs is that they can design and train deeper CNN models. However, increasing the number of convolutional layers in the graph by GCNs may cause the gradient disappearance, which means that smoothing occurs during backpropagation, i.e., the features of all nodes in the graph converge to the same value. Thus, GCNs are generally shallow structures that contain 2–3 graph convolutional layers. Shallow structures limit the performance of the model, because they cannot mine higher-order node information. Gradient disappearance poses a challenge for the deep GCNs designing.

Gradient disappearance is also a significant factor limiting the training of deep CNNs models. ResNet introduces residual connections between convolutional layers to construct deep CNNs. Residual connections can avoid the gradient disappearance problem well by constantly reusing features. DenseNet (Huang et al., 2017) further expands ResNet, which introduces more connections between convolutional layers. However, as the convolutional layer increases, pooling can lead to more spatial information loss, but the convolution proposed in literature (Yu and Koltun, 2015) solves this problem. The above concepts have driven the rapid development of CNNs, and if they are introduced into GCN_S_, whether the model can get similar results to CNNs?

DeepGCNs use ResNet, DenseNet, and Void Convolution to train deep neural networks in computer vision to get success in point cloud semantic segmentation filed. However, DeepGCNs are a kind of deep neural networks based on single-feature training. Because graph convolutional networks based on single feature training cannot fully depict the relevant characteristics of the graph, and Zhao et al. (2023) consider the interaction between features and propose a GCN based on dual feature interaction. Therefore, HDGCN adds semantic features on top of structural features, which not only enriches the diversity of graph information but also enhances node features. However, HDGCN is a shallow neural network that can only demonstrate excellent performance on simple tasks, and cannot learn higher-level features for more abstract and complex data. Therefore, it is necessary to deepen the algorithm.

Drawing on the successful experience of using residual connections to construct deep GCNs in DeepGCNs, this paper successfully constructs deep dual-channel Graph Convolutional Networks Based on Hybrid Features (D2GCN) using residual connections in HDGCNs. This paper shows how residual connections can be combined with multi-layer graph convolution operations to construct D2GCN, and the effect of residual connections on the accuracy and stability of D2GCN is analyzed. This paper applies D2GCN to the task of node classification, the number of neural network layers can reach 16, and the performance of D2GCN on the three datasets is improved by about 3% compared with SOAT.

In summary, the main contributions of this paper are as follows:

1. Most of the existing graph neural networks are implemented based on single channel neural networks, and few achievements of dual-channel graph neural networks have been published. However, the relevant achievements of dual-channel deep graph neural networks have not been published so far. This paper is the first academic paper discussing dual-channel deep graph neural networks. The algorithm proposed in this paper is verified and introduced by many measure approaches, such as theory, experiment, comparative analysis parameter sensitivity etc.
2. To make full use of the feature diversity and complementarity on graph, this paper fuses the text features and structural features into hybrid features, which enriches the information diversity on graph and enhances the feature expression ability of the nodes.
3. Based on text features, structural features and hybrid features, three kinds of variation models based on D2GCN are proposed by using residual networks, such as D2GCN_(structure)_, D2GCN_(semantic)_ and D2GCN_(hybrid)_. D2GCN is only the general name of dual-channel deep graph neural network, and D2GCN_(structure)_, D2GCN_(semantic)_, and D2GCN_(hybrid)_ determine the type of graph features placed in the neural network channel.

## 2 Related words

Since graphs are ubiquitous in the real world, researches on graphs are receiving more and more attention from researchers. Graphs have been widely used to represent various domain information, such as recommendation system (Monti et al., 2017b), molecular graph structure (Wale et al., 2008; Zitnik and Leskovec, 2017), social network (Armeni et al., 2017), and Linguistics (Bastings et al., 2017; Marcheggiani and Titov, 2017). Graphs have also played a key role in deep learning, such as classifying the role of a protein on a bio-interaction graph, predicting the role of an author in a cooperative network, recommending new friends to users in a cooperative network, recommending new friends to users in social networks, recommending new friends to users in social networks, and recommending ads to users etc. However, most traditional deep learning models, such as convolutional neural networks (CNNs) and recurrent neural network (RNNs), process data limited to Euclidean space and have translational invariance and local connectivity, such as images and text. As irregular non-European data, CNNs and RNNs cannot be directly applied to the field of graph. The challenge of deep learning of graphs lies in encoding the high-dimensional, non-Euclidean information into the form of embedding and input them into subsequent analysis tasks. Graph Convolutional Neural Networks (GCNs) provide a novel direction for processing graph data, for example, graphs are used to represent individuals and the connections between individuals in social networks, and then high irregular graph data in non-European spaces are obtained. GCNs can assess the strength of individual connections in social networks, and get more accurate evaluation between individuals (Tang and Liu, 2009). GCNs have many applications in the field of computer vision, for example, graphs are used to represent semantic relationships between objects, and then objects are detected and segmented, semantic relationships between objects are predicted (Qi et al., 2017; Xu et al., 2017; Li Y. et al., 2018; Yang et al., 2018) at last. Human joints can be represented by graph and then GCNs is used to recognize the actions in video (Jain et al., 2016; Yan et al., 2018). GCNs are also the perfect approach for dealing with 3D point clouds due to its non-structural properties (Chen and Zhang, 2023; Jiang et al., 2023; Khodadad et al., 2023; Wang L. et al., 2023). Similarly, GCNs also have many applications in the field of natural language processing. In terms of sentiment analysis, they are not only applicable to unimodal sentiment analysis (Zhang et al., 2022) but also to multimodal sentiment analysis (Firdaus et al., 2023). For example, Huang et al. (2023) propose CRF-GCN, a model that utilizes conditional random fields (CRF) to extract opinion scopes of specific aspect words and integrates their contextual information into global nodes. These global nodes are then introduced into GCNs to effectively address the issue of fluctuating model accuracy in sentences with multiple aspect words.

Current GCNs algorithms can be divided into two categories: spectral-based and spatial-based methods. Bruna proposed the Spectrum CNN based on the convolutional theorem (Bruna et al., 2014) in 2014, which imitates the characteristics of convolutional neural networks by superimposing multi-layer graph convolutions, and defines convolutional kernels and activation functions for each layer, and form graph convolutional neural networks. Due to its high spatiotemporal complexity, Defferrard subsequently proposed ChebNet (Defferrard et al., 2016) in 2016 to reduce the temporal complexity by using the Chebyshev polynomial as a convolutional kernel. Due to the high complexity of eigenvalue decomposition of Laplace matrices, David and Hammond (2011) uses K-order truncation of Chebyshev polynomials instead of convolutional kernels, converts the modeling range of convolutional kernels from the entire graph to the K-order neighbors of the nodes, and reduces the number of parameters of convolutional kernels. Kipf and Welling (2017) proposes a hierarchical propagation method using a first-order approximation ChebNet, where each graph convolutional layer aggregates only first-order neighbors, and multiple graph convolutional layers can share a convolutional kernel, which can significantly reduce the number of parameters. With the increase of the number of layers, more information can be aggregated from distant neighbors. These methods are all defined in the perspective of the spectral features, while the spatial-based method appears earlier and it is more popular at present.

The core idea of the spatial-based approaches is to iteratively aggregate the features of neighbor nodes by defining aggregation functions, and then to update the features of the current nodes. In 2009, Gori proposed GNNs (Scarselli et al., 2009) method, which uses circular recursive functions as aggregate functions, and each node updates its own embedding by aggregating neighbor node information. In 2016, DCNN (Atwood and Towsley, 2016) regarded graph convolution as a diffusion procedure, and the information between nodes spreads with a certain probability, In 2017, Hamilton proposed GraphSAGE (Hamilton et al., 2017) method, which gives three aggregation functions to update the node state, such as mean aggregation, LSTM aggregation and pooling aggregation. Gilmer find that all spatial-based graph convolutional networks aggregate neighbor’s state in some form to update the state of central node, so a framework MPNN (Gilmer et al., 2017) of spatial-based graph convolution is proposed for predicting chemical molecular properties. Under the inspiration of spectral-based graph convolutional network, the spatial-based graph convolutional network quickly become popular, and begin to develop toward a unified framework.

Nowadays, many scholars have solved numerous problems based on GCN. Li et al. (2023) propose DMRGCN, a novel bidirectional mutually reinforcing GCN, which investigates the semi-supervised node classification problem under noisy labels. Wang K. et al. (2023) propose mGNN, which extends the imbalanced classification concept in the field of machine learning to graph structures and effectively improves the classification performance of graph neural networks. Zhu et al. (2023) propose RGCNU, which maps the relationship between noisy monitoring data and uncertain residual life. Hou et al. (2021) propose ST Trader, which first uses VAE to reduce the dimensions of stock related information and convert it into a graph structure. Then, GCN-LSTM is used to effectively predict stock movements. Despite the rapid development has got for GCNs, most of the GCNs are shallow structures. At present, some researchers have begun to train deep GCNs using different methods. GraphSAGE simultaneously uses node feature and structural feature to obtain graph embeddings, which is more scalable. In 2017, Pham proposed CLN (Pham et al., 2017) for relational classification, where model performance peaks when the depth of CLN reaches 10 layers, and model performance decreases as the increasing depth of CLN. In 2018, Rahimi et al. (2018) used GCN to integrate user text with network structures to achieve a more accurate geolocation of social media users. However, the authors find that the model performance gradually decreases when the depth of the Highway GCN is 6. Xu et al. (2018) proposes Jumping Knowledge Networks, which adjusts the range of the aggregated features according to different positions and structures of each node on graph, and the model is also limited to a six-layer structure. The number of graph convolutional layers limits the performance of the above GCNs, for example, the 10-layer graph convolution is superimposed, the model performance would decrease. In 2018, Li Q. et al. (2018) found that the biggest obstacle to training deep GCNs was over-smooth, and other research results (Zhou et al., 2018; Wu et al., 2019) also proved that the convolution operation of multi-layer graphs would lead to vanishing gradient. In order to alleviate the occurrence of over-smoothing phenomena, Li proposed DeepGCNs in 2019, which adds residual/dense connections to train deep GCNs inspired by deep CNNs, such as ResNet, DenseNet, etc. Klicpera is based on the intrinsic connections between GCNs and PageRank (Page et al., 1998; Klicpera et al., 2019a), it designs a propagation scheme based on personalized PageRank. In 2020, Rong and Zhao proposed DropEdge (Rong et al., 2019) and PairNorm (Zhao and Akoglu, 2020), respectively to migrate Dropout and BatcheNorm to GCNs, which can also obtain better embedding and classification effects. Zhang et al. (2023) propose DRGCN, which utilizes dynamic block initialization information and employs evolution blocks to model the residual evolution patterns between layers. This approach effectively alleviates the over-smoothing issue in deep GCNs. Yang et al. (2023) propose EM-GCN, a model that introduces the expectation–maximization algorithm and utilizes approximate inference to overcome excessive smoothing in topology optimization for any GCN.

The approaches of making graph convolutional neural networks deeper can also be achieved through some alternative methods, for instance, we can design a high-order graph convolutional neural networks. Therefore, many researchers have adopted a shallow alternative method, it is that the GCNs consider higher-order neighbors in single-layer graph neural networks, for example, k-GNNs uses high-order Weisfeiler-Lehman for designing the graph neural networks (Morris et al., 2019), the MixHop solves the mixing problem of neighboring features at different distances (Abu-El-Haija et al., 2019), and GDC (Klicpera et al., 2019b) enhances the performance of graph neural networks using graph diffusion. These high-order neural networks can usually obtain better embedding and classification effects, however, the above research is only available for deep neural networks based on single-feature training. For the complex information on graph, the deep structure of the current graph neural network using single feature cannot completely reveal the complex information of the graph. In order to reflect the diversity of the graph and avoid the over-smoothing phenomenon caused by enlarging receptive field of the graph convolutional networks, this paper uses residual networks to construct a dual-channel deep graph convolutional network based on hybrid features of the graph.

## 3 Preliminaries

### 3.1 Graph convolutional network

In 2017, Kipf further proposed graph convolution with K-order Laplace polynomials as follows (Kipf and Welling, 2017):


(1) $$Ug_{\theta }\left(\Lambda \right)U^{T}x\approx U\left(\overset{K}{\underset{𝓁=0}{\sum }}\theta_{𝓁}\Lambda^{𝓁}\right)U^{T}x=\left(\overset{K}{\underset{𝓁=0}{\sum }}\theta_{𝓁}L^{𝓁}\right)x\text{.}$$

Here, $\theta \in R^{K+1}$ is a polynomial coefficient vector. If K=1 , $\theta_{0}=2\theta$ , and $\theta_{1}=-\theta$ , we can get a new convolution operation $g_{\theta }*x=\theta \left(I+D^{-1/2}AD^{-1/2}\right)x$ . Through the renormalization tricks, the GCN will replace the expression $I+D^{-1/2}AD^{-1/2}$ with $\tilde{P}={\tilde{D}}^{-1/2}\tilde{A}{\tilde{D}}^{-1/2}={\left(D+I_{n}\right)}^{-1/2}\left(A+I_{n}\right){\left(D+I_{n}\right)}^{-1/2}$ , and it gets a graph convolutional layer as follows:


(2) $$H^{\left(𝓁+1\right)}=\sigma \left(\tilde{P}H^{\left(𝓁\right)}W^{\left(𝓁\right)}\right)\text{.}$$

The σ is the activation function ReLU. Nodes aggregate high-order neighbor node information in GNNs, it would cause that nodes become indistinguishable with other nodes, and there exists a gradient vanishing during backpropagation. In order to avoid over-smoothing and gradient vanishing, DeepGCNs use the method adopted by deep CNNs to construct the deep structure of GCNs.

### 3.2 DeepGCNs

In 2019, Li introduced the method of training deep CNNs as ResNet, DenseNet, and dilated convolutions to propose DeepGCNs. DeepGCNs added the residual/dense network and dilated convolutions based on GCNs (Li et al., 2019).


(3) $$H^{\left(𝓁+1\right)}=\sigma \left(\tilde{P}H^{\left(𝓁\right)}W^{\left(𝓁\right)}\right)+H^{\left(𝓁\right)}\text{.}$$

DeepGCNs use the information flow of different graph convolutional layers to reuse features between graph convolutional layers by dense connections.


(4) $$H^{\left(𝓁+1\right)}=\mathrm{Concat}\left(\begin{array}{l} \sigma \left(\tilde{P}H^{\left(𝓁\right)}W^{\left(𝓁\right)}\right),\sigma \left(\tilde{P}H^{\left(𝓁-1\right)}W^{\left(𝓁-1\right)}\right),\cdots ,\\ \sigma \left(\tilde{P}H^{\left(0\right)}W^{\left(0\right)}\right),H^{\left(0\right)} \end{array}\right)\text{.}$$

DeepGCNs obtain the surrounding nodes of the target node when the dilated rate *d* is determined by Dilated KNN:


(5) $$N^{\left(d\right)}\left(v\right)=u_{1},u_{1+d},\cdots ,u_{1+\left(k-1\right)\times d}\text{.}$$

$\left(u_{1},u_{2},\cdots ,u_{k\times d}\right)$ is an ordered set of k×d nearest neighbors, When the dilated rate is *d*, $\left(u_{1},u_{1+d},\cdots ,u_{1+\left(k-1\right)\times d}\right)$ is the neighbor node of the node v . Since GCNs and DeepGCNs are single feature graph convolutional networks, and the graph contains much complex information, so the text features are integrated based on the structural features of the graph to enhance the embedding ability, which meets the diversity of graph information.

### 3.3 HDGCN

In 2021, Li (Yang et al., 2018) proposes HDGCN, which uses a dual channel GCNs structure to jointly model the structure and semantic features of nodes, complementing and enhancing the features of nodes as follows.


(6) $$H_{t}^{\left(𝓁+1\right)}=\sigma \left(P_{t}H_{t}^{\left(𝓁\right)}W_{t}^{\left(𝓁\right)}\right)\text{.}$$

$\forall t\in \left\{1,2,\cdots ,T\right\}$ , the output matrix is $H_{t}\in R^{N_{t}\times d}$ , parameter matrix is $W_{t}\in R^{d\times d}$ .

Because the semantic features of the nodes contain the semantic features of weakly correlated neighboring nodes, it will become noise data that affects the training effect of the model. In order to reduce the noise interference on model training and enhance the feature embedding ability, the dual-channel GCN introduces Graph Attention Network and Gateway Recurrent Unit as follows:


(7) $$z_{\mathrm{GAT}}^{v}=\alpha_{v}h_{v}\text{,}$$

(8) $$z_{GRU}^{v}=g_{v}⊙h_{v}\text{.}$$

Attention coefficient is


(9) $$\alpha_{v}=\frac{\exp\left(e_{v}\right)}{\underset{v\in V}{\sum }\exp\left(e_{v}\right)}\text{.}$$

The relevance is $e_{v}=a^{T}\cdot Relu\left(W\cdot h_{v}+b\right)\text{,}$ the gating embedding is $g_{v}=\mathrm{Sigmoid}\left(W\cdot h_{v}\right),\forall v\in \left\{1,2,\cdots N\right\}\text{.}$ The $a^{T}$ , W , and b are attention embeddings, weight parameters, and biases, respectively. According to the importance of each node, we give it a corresponding weight to reflect its importance. To improve the accuracy of graph convolutional networks based on hybrid feature, we expand the receptive field and aggregate the features of the higher-order neighboring nodes. To avoid over-smoothing, we also adopt the residual networks.

Since the text features and structural features have different influences on the model during training, three kinds of dual-channel GCNs models are proposed as follows.

## 4 Dual channel graph convolutional neural network framework

In this paper, the D2GCN is proposed, which aims at solving the fact that current GNNs cannot fully mine the high-order features based on dual-channel GNNs. In D2GCN, hybrid features are used as the inputs, and the feature matrix of each layer is reused for residual networks to avoid gradient vanishing in backpropagation. To enhance the feature embedding ability of nodes, the upstream node features are con-catenated as the input of the fully connected layer.

### 4.1 Definition

Graphs G can be represented by the triplets $G=\left(v,\varepsilon_{1},\varepsilon_{2},\phi ,\varphi \right)$ , where V are set of unordered nodes, $\varepsilon_{1}$ and $\varepsilon_{2}$ represent the edge sets of semantic networks and structural networks, $\phi :\varepsilon_{1}\to R_{1}$ is a mapping function based on the node position relationships, $\varphi :\varepsilon_{2}\to R_{2}$ is a mapping function based on the text content between nodes, $R_{1}$ and $R_{2}$ are a collection of node relationship types. A and D are adjacency matrix and degree matrix, respectively. $X\in R^{n\times d}$ is the feature matrix of the node, each of these nodes corresponds to a d dimension feature embedding $X_{v}$ . Regularized Laplace matrices are semi-definite symmetric matrix, defined as $L=I_{n}‐D^{-1/2}AD^{-1/2}$ . Its eigenvalues are decomposed into $U\Lambda U^{T}$ . Λ is a eigenvalues diagonal matrix of L , $U\in R^{n\times n}$ is a unitary matrix composed of eigenvectors of L .The graph convolution operation between the input signal x and the filter $g_{\gamma }\left(\Lambda \right)=\mathrm{diag}\left(\gamma \right)$ is defined as $g_{\gamma }\left(L\right)*x=Ug_{\gamma }\left(\Lambda \right)U^{T}x$ , $\gamma \in R^{n}$ is the coefficient vector corresponding to the filters.

The following three networks are the data basis of modeling in this paper, so the following explanations are given as follows:

Semantic network: semantic network refers to a network composed of texts relationships. If two nodes have the same words in their text features, a new edge will be added to these two nodes.
Structure network: structure network is the original network structure, in which no new nodes and edges are added.
Hybrid network: semantic network and structural network are combined to form a hybrid network.

### 4.2 Dual-channel deep graph convolutional neural network

#### 4.2.1 D2GCN based on structural features [D2GCN _(structure)_]

**Figure 1:**
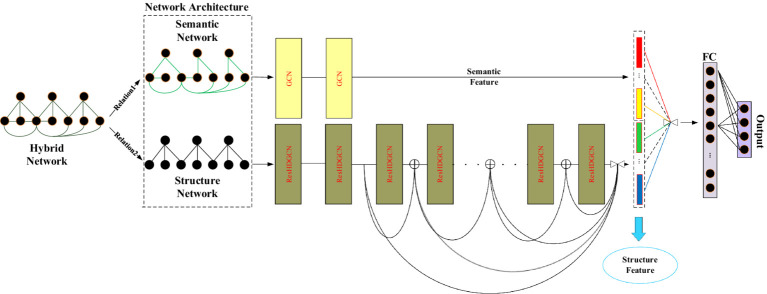
Illustration of D2GCN based structural features (“ ⊕ ” represents addition operation, which means element-wise addition; “
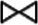
” represents vector concatenation).

The framework of D2GCN based on structural features is shown in Figure 1.

In this model, the semantic network is trained by shallow neural network, and the structural network is trained by deep neural network. Finally, the two different embeddings are integrated.

The graph based on hybrid features is used as the input of the model, and the input is divided into two single feature graphs through the dual-channel GCNs: graph based on structural features and graph based on text features, and node embeddings of two different types of features is obtained. Because the graph based on text features may contain many weak correlations between nodes, it becomes noise that affects the performance of the model, and the shallow structure of the graph convolutional networks only aggregates the neighboring node features. Therefore, the text features of the node are used as the input of the shallow structure of the graph convolutional network. In addition, the graph based on the structural features is used as the input of the deep graph convolutional networks, because the deep graph convolutional networks can aggregate high-order features. When the scale of the graph convolutional network aggregation is gradually expanded to all nodes of the graph, the features of the node will become indistinguishable, causing the gradient vanishing during the period of backpropagation. With the increase of the graph convolutional layer, the output of the graph convolutional layer is repeatedly reused using residual networks to ensure the difference, the D2GCN_(structure)_ can be formulated as follows:


(10) $$H_{\left(\mathrm{structure}\right)}^{\left(l+1\right)}=\sigma \left(\tilde{P}H_{\left(\mathrm{structure}\right)}^{\left(l\right)}W^{\left(l\right)}\right)+H_{\left(\mathrm{structure}\right)}^{\left(l\right)}\text{.}$$

According to theoretical analysis, the main reason for the complexity of the D2GCN_(structure)_ model is the residual connection of the structural network. The main reason for the complexity of the D2GCN_(structure)_ model is the residual connection of the structural network. According to formula 10, the complexity of $\tilde{P}H_{\left(\mathrm{structure}\right)}^{\left(l\right)}W^{\left(l\right)}$ is $Ο\left({\left|V\right|}^{3}\right)$ , where *V* is the number of network nodes and the complexity of $H_{\left(\mathrm{structure}\right)}^{\left(l\right)}$ is $Ο\left(\left|V\right|\right)$ . Therefore, the time complexity of D2GCN_(structure)_ is $Ο\left({\left|V\right|}^{3}\right)$ .

#### 4.2.2 D2GCN based on semantic features [D2GCN _(semantic)_]

**Figure 2:**
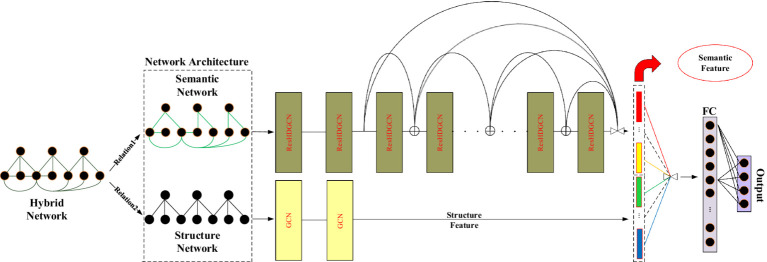
Illustration of D2GCN based semantic features (The symbol interpretation in this section is the same as in Figure 1).

The framework of D2GCN based on semantic features is shown in Figure 2.

In this model, the structural network is trained by shallow neural network, and the semantic network is trained by deep neural network. Finally, the two different embeddings are integrated.

For large-scale sparse graphs, nodes belonging to the same type may not have a neighboring relationship or even a weak correlation. However, there is a greater probability that a node belongs to the same type as its neighbors. Thus, the features around the nodes are aggregated by the shallow structure of the graph convolutional networks. Because the graph based on text features is more dense than the graph based on structural features, many nodes of the same type without edges in the sparse graph based on structural features may establish direct/indirect connections in D2GCN_(semantic)_. Therefore, the graph convolution operation is used repeatedly to obtain the global structure based on the text feature graph. The local structure and global structure of the graph are fused to improve the accuracy of the downstream classification tasks. The specific formula is as follows:


(11) $$H_{\left(se\mathrm{mantic}\right)}^{\left(l+1\right)}=\sigma \left(\tilde{P}H_{\left(\mathrm{semantic}\right)}^{\left(l\right)}W^{\left(l\right)}\right)+H_{\left(\mathrm{semantic}\right)}^{\left(l\right)}\text{.}$$

Similar to the complexity analysis of D2GCN_(structure)_, the time complexity of D2GCN_(semantic)_ is $Ο\left({\left|V\right|}^{3}\right)$ .

#### 4.2.3 D2GCN based on hybrid features [D2GCN _(hybrid)_]

**Figure 3:**
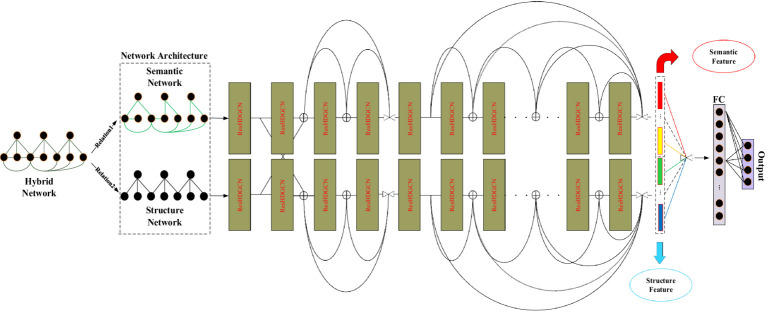
Illustration of D2GCN based hybrid feature (The symbol interpretation in this section is the same as in Figure 1).

The framework of D2GCN based on hybrid features is shown in Figure 3.

In this model, the structural network is trained by deep neural network, and the semantic network is also trained by deep neural network. Finally, the two different embeddings are integrated.

The probability that the interconnected nodes in the sparse graph belong to the same type decreases with the increase of distance, so the output of the shallow structure of the residual networks that reuses graph convolutional neural network is used to obtain the local graph structure based on text or structural features. The cross-reuse of text features and structural features enables features to complement each other and enhance the embedding ability of node features. The specific formula is as follows:


(12) $$\begin{array}{l} H_{\left(\mathrm{semantic}\right)}^{\left(1\right)}=\sigma \left(\tilde{P}H_{\left(\mathrm{semantic}\right)}^{\left(0\right)}W^{\left(0\right)}\right)+H_{\left(\mathrm{semantic}\right)}^{\left(0\right)},H_{\left(\mathrm{structure}\right)}^{\left(1\right)}\\ \;=\sigma \left(\tilde{P}H_{\left(\mathrm{structure}\right)}^{\left(0\right)}W^{\left(0\right)}\right)+H_{\left(\mathrm{structure}\right)}^{\left(0\right)}\text{,} \end{array}$$

(13) $$\begin{array}{l} H_{\left(\mathrm{semantic}\right)}^{\left(2\right)}=H_{\left(\mathrm{structure}\right)}^{\left(1\right)}+H_{\left(\mathrm{semantic}\right)}^{\left(2\right)},H_{\left(s\mathrm{tructure}\right)}^{\left(2\right)}\\ \;=H_{\left(\mathrm{semantic}\right)}^{\left(1\right)}+H_{\left(\mathrm{structure}\right)}^{\left(2\right)}\text{.} \end{array}$$

Then the deep graph convolutional network is constructed by the residual network, and the local structure of the graph obtained by the convolutional network of the previous layer of graph is used as input to obtain the global structure of the graph. Finally, the global structure of the two features is fused to obtain a probability matrix based on the hybrid features $H_{\left(\mathrm{hybrid}\right)}^{\left(l+1\right)}$ . The specific formula is as follows:


(14) $$H_{\left(\mathrm{semantic}\right)}^{\left(l+1\right)}=\sigma \left(\tilde{P}H_{\left(\mathrm{semantic}\right)}^{\left(l\right)}W^{\left(l\right)}\right)+H_{\left(\mathrm{semantic}\right)}^{\left(l\right)},l\geq 2\text{,}$$

(15) $$H_{\left(\mathrm{structure}\right)}^{\left(l+1\right)}=\sigma \left(\tilde{P}H_{\left(\mathrm{structure}\right)}^{\left(l\right)}W^{\left(l\right)}\right)+H_{\left(\mathrm{structure}\right)}^{\left(l\right)},l\geq 2\text{,}$$

(16) $$H_{\left(\mathrm{hybrid}\right)}^{\left(l+1\right)}=\mathrm{concat}\left(H_{\left(\mathrm{semantic}\right)}^{\left(l+1\right)},H_{\left(\mathrm{structure}\right)}^{\left(l+1\right)}\right),l\geq 2\text{,}$$

(17) $$H_{\left(\mathrm{hybrid}\right)}^{\left(l+1\right)}=\sigma \left(\tilde{P}H_{\left(\mathrm{hybrid}\right)}^{\left(l\right)}W^{\left(l\right)}\right)+H_{\left(\mathrm{hybrid}\right)}^{\left(l\right)}\text{.}$$

Due to the fusion structure of D2GCN _(hybrid)_, both channels are residual connected, resulting in a time complexity of $Ο\left(2{\left|V\right|}^{3}\right)$ and simplified to $Ο\left({\left|V\right|}^{3}\right)$ .

## 5 Experimental results and analysis

This section may be divided by subheadings. It should provide a concise and precise description of the experimental results, their interpretation, as well as the experimental conclusions that can be drawn.

### 5.1 Datasets

To assess the effectiveness of D2GCN, this paper uses three reference network datasets, such as CiteSeer, DBLP, and SDBLP. The statistics of the datasets are shown in Table 1. Each dataset is divided into semantic networks and structural networks. In the semantic network, the edge connection relationship between nodes is constructed according to the word co-occurrence, if the same word appears in the text of each node, there is an edge link between nodes. In the structural network, the relationship between nodes is determined according to the citation relationship between different documents. SDBLP is a simplified dataset of DBLP, in which nodes with less than 3 references are deleted, it is that nodes with node degree less than 3 will be deleted.

**Table 1:** Dataset description.

Dataset	CiteSeer		SDBLP		DBLP	
	Structure	Semantic	Structure	Semantic	Structure	Semantic
Node	4,610	4,610	3,119	3,119	17,725	17,725
Edge	5,923	819,346	39,516	439,182	105,781	125,360
Training	1,333	1,333	666	666	3,333	3,333
Validation	667	667	334	334	1,667	1,667
Test	2,610	2,610	2,119	2,119	12,725	12,725

### 5.2 Baselines

This paper compares D2GCN with the following baseline methods designed to generate node embedding:

DeepGCNs_(structure)_: this model inputs the structural features to Current DeepGCNs.
DeepGCNs_(semantic)_: this model inputs the semantic features to Current DeepGCNs.
D2GCN_(JKNet)_: the use of initial residuals and identity mappings can solve the problem of over-smooth. In each layer, the initial residuals construct a jump connection from the input layer, while the identity map adds the identity matrix to the weight matrix. When increasing the depth of the model, these two techniques can prevent over-smooth and continuously improve the performance of the model.
D2GCN_(Drop)_: it proposes a random removal edge strategy using DropEdge by a certain ratio, DropEdge increases the diversity of the inputs to prevent overfit, and alleviate over-smooth.
HDGCN: it is similar to the dual-channel deep graph neural network based on hybrid features proposed in this paper, but the depth of the model is only two layers, which is a shallow dual channel graph neural network.
D2GCN_(structure)_: the dual-channel deep graph convolutional neural network proposed in this paper only considers to model the structural features of structure network.
D2GCN_(semantic)_: the dual-channel deep graph convolutional neural network proposed in this paper only considers to model the text features of semantic network.
D2GCN_(hybrid)_: the dual-channel deep graph convolutional neural network proposed in this paper considers to model the hybrid features integrated by the structural and semantic features.

### 5.3 Semi-supervised node classification

This paper compares D2GCN with the following baseline methods designed to generate node embedding.

For the semi-supervised node classification task, we randomly divide datasets into training/validation/test on CiteSeer, SDBLP, and DBLP datasets. This paper applies two Deep GNN models, which are GCNII and DropEdge, to HDGCN and GCN. We use Adam SGD as an optimizer to train D2GCN, and we set learning rate as 0.01, iterations as 200, dropout ratio as 0.5, weight decay rate as 0.0005, and embed dimension as 64. In the experiment, this paper uses D2GCN to train hyper-parameters of the model, and divides the training set into several small batches of data to update the parameters. In order to reduce the error, this paper repeats the experiment for 10 times, and the average value of the accuracy is shown in Table 2.

**Table 2:** Node classification accuracy with various depts.

Dataset	Model	Layers			
		2	4	8	16
CiteSeer	Deep GCNs _(structure)_	77.63	78.12	78.36	77.74
	Deep GCNs _(semantic)_	67.45	67.33	66.51	18.58
	D2GCN _(JKNet)_	81.99	85.13	86.63	85.71
	D2GCN _(Drop)_	83.49	85.33	85.17	84.64
	HDGCN	82.30	-	-	-
	D2GCN _(structure)_	84.15	84.55	85.48	83.41
	D2GCN _(semantic)_	84.15	84.36	84.55	84.95
	D2GCN _(hybrid)_	**84.17**	**86.43**	**86.77**	**86.99**
SDBLP	Deep GCNs _(structure)_	82.41	82.21	82.51	80.15
	Deep GCNs _(semantic)_	70.03	65.08	34.50	27.70
	D2GCN _(JKNet)_	81.31	81.55	82.23	82.21
	D2GCN _(Drop)_	81.93	82.40	81.69	82.11
	HDGCN	81.50	-	-	-
	D2GCN _(structure)_	**81.81**	**82.14**	**82.36**	**83.47**
	D2GCN _(semantic)_	81.78	82.36	82.47	83.11
	D2GCN _(hybrid)_	81.62	81.97	82.17	82.93
DBLP	DeepGCNs _(structure)_	80.70	79.91	79.12	79.80
	DeepGCNs _(semantic)_	67.42	60.70	44.57	44.78
	D2GCN _(JKNet)_	80.46	79.03	79.76	79.01
	D2GCN _(Drop)_	80.42	80.65	80.65	80.28
	HDGCN	80.28	-	-	-
	D2GCN _(structure)_	80.37	80.17	78.29	78.10
	D2GCN _(semantic)_	**80.09**	**80.33**	**80.61**	**80.88**
	D2GCN _(hybrid)_	80.55	80.30	80.18	80.21

The data in bold in table represents all the results of the best model corresponding to a certain dataset.

As shown in Table 2, the convolution operation of D2GCN_(hybrid)_ at 2, 4, 8, and 16 layers obtains the optimal values compared with the baseline methods on the CiteSeer dataset, and the model performance increases with the increasing number of graph convolutional layers. The model performs best when the graph convolutional layer reaches 16. The baseline methods shows that the model performance first increases and then decreases with the increasing number of convolutional layers for the dual-channel deep GCNs, resulting in little significance for the deep structure of the model based on hybrid features.

Since the SDBLP is a dense network, the probability that the central node and its surrounding nodes belong to the same type decreases as the increasing distance between the nodes. Therefore, the shallow GCN performs best in two-layer graph convolutional operation compared to D2GCN_(structure)_, and the text features contain many weakly correlated features, which aggregate the information of the surrounding node as noise data to interfere center node, and classify the center node and its unrelated nodes into the same category. Therefore, D2GCN_(structure)_ is better classified than D2GCN_(semantic)_. However, the GCN has over-smooth phenomenon and the model performance has gradually decreased with the increasing layer of the graph convolution. Conversely, D2GCN_(structure)_ aggregates the features of high-order neighboring nodes, and with the increase of convolutional layers, the accuracy of the model in the classification task is continuously improved. The other baseline methods show a trend that the accuracy first rises and then decreases with the increase of the convolutional layers. The performance of D2GCN_(structure)_ is the best when the depth of graph convolution operation is 16.

On the DBLP, deep graph convolutional neural network D2GCN_(semantic)_ based on text feature is better than deep graph convolutional neural network D2GCN_(structure)_ based on structural features. Because the number of neighboring nodes of the central node is smaller, but the probability that they belong to the same type is higher. Therefore, the baseline methods are better than the proposed D2GCN when the number of convolution layer is 2. However, D2GCN also increases model performance as the increasing number of graph convolutional layers. Other baseline methods show a tendency that the accuracy first rises and then decreases in the classification task, or show an unstable phenomenon of alternating ascent and descent.

### 5.4 Visualization

**Figure 4:**
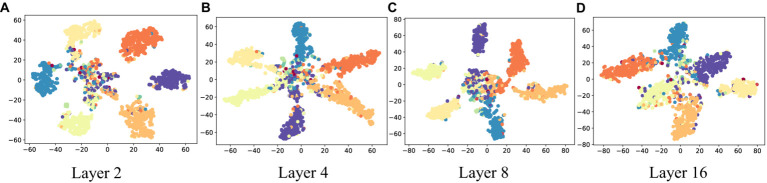
Visualizations of node representations with different numbers of layers on CiteSeer. **(A)** Layer 2; **(B)** Layer 4; **(C)** Layer 8; **(D)** Layer 16.

To further illustrate the effectiveness of D2GCN, this paper conducts a set of visualization experiments. We use t-SNE to map the embeddings of nodes into 2D space on CiteSeer, the embeddings of different depths obtained through D2GCN are shown in Figure 4, and different colors represent to different node label. Through visual experiments, it can be seen that the D2GCN gets better and better as the increasing number of convolutional layers. Specifically, the internal similarity becomes higher and higher, and the boundaries between different node labels are clearer as the increasing depth of the model.

### 5.5 Hyperparameter analysis

**Figure 5:**
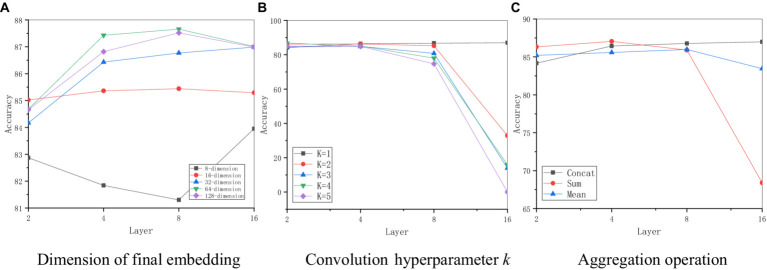
Parameter sensitivity of D2GCN. **(A)** Dimension of final embedding. **(B)** Convolution hyperparameter *k*. **(C)** Aggregation operation.

This paper performs a sensitivity analysis for some main hyperparameters in D2GCN, as shown in Figure 5.

#### 5.5.1 Final embedding dimension *F*

This paper first tests the effect of the final embedding dimension *F*, as shown in Figure 5A, when the embedding dimension is 32, the model performance increases with the increase of the graph convolutional layer. When the embedding dimension is 8, the performance decreases as the increasing number of convolutional layers. And then when the convolutional layer increases to 16 layers, the model performance improves best. Model performance of other embedding methods improves with the increase of graph convolutional layers, and then the model performance decreases when the 16-layer graph convolutional operation is performed. When the dimension is 32, the model performs best in the node classification task.

#### 5.5.2 Convolution hyperparameter *K*

In this paper, the influence of the *K*-order approximation of the local spectral filter on the model is studied, as shown in Figure 5B, the model performs stably in the classification task with the increase of the graph convolutional layer. When the graph convolutional layer is 8, the model performance begins to decrease as the increases of *K*. When the graph convolutional layer reaches 16, the model performance drops significantly, and at that time, the model performance drops the fastest. However, when the hierarchical convolution operation is performed, the model performance increases with the increase of the graph convolutional layer, and when the graph convolutional layer is 16, the model performance reaches the best. Therefore, D2GCN, restricts hierarchical convolution operations.

#### 5.5.3 Feature aggregation operation

In this paper, different feature aggregation operations are also studied, as shown in Figure 5C, the model performance is improved fast where the embeddings of semantic and structural networks are concatenated. However, the model uses the fusion approach of averaging and summing methods to lead to performance degradation as the increasing number of convolutional layers, when the number of graph convolutional layer is 16, the model performance decreases the most, especially the summing method. The simple concatenation operation continuously improves the model performance as the increasing number of graph convolutional layers, and the model performs best when the number of graph convolutional layer reaches 8. When the number of convolutional layer reaches 16, its performance is the best. Therefore, this paper uses concatenation method to fuse semantic features and structural features.

## 6 Conclusion

In this paper, a dual-channel deep graph convolutional neural network (D2GCN) based on hybrid features is proposed. According to the text features, the residual connection is used to construct the deep graph convolution neural network, and for the structural features of the graph, the two-layer graph convolution neural network is used, which is the D2GCN_(semantic)_. D2GCN_(structure)_ trains a deep graph convolutional neural network with the structural features of the graph, in contrast, the shallow structure of the graph convolutional neural network is trained based on the text features of the graph. In this paper, D2GCN_(structure)_ is constructed using residual networks, and the two features of the graph are fused by concatenating strategy. D2GCN_(hybrid)_ uses the text features and structural features of the graph to simultaneously train a dual-channel deep graph convolutional neural network constructed by residual networks, in which the output of the graph convolutional neural network is cross-reused, so that the two features complement each other and improve the performance of the model in the node classification task. The experimental results in this paper demonstrate the effectiveness of D2GCN in node classification task. As an efficient way to improve model performance, it is a potential research how to incorporate pre-training into D2GCN.

## Data availability statement

Publicly available datasets were analyzed in this study. This data can be found here: http://www.cs.umd.edu/~sen/lbc-proj/data/cora.tgz; http://www.cs.umd.edu/~sen/lbc-proj/data/citeseer.tgz.

## Author contributions

ZY: Writing – original draft, Data curation, Methodology, Validation. ZL: Writing – original draft. GL: Writing – review & editing. HZ: Supervision, Writing – review & editing.
